# Shear Bond Strength of Resin Luting Materials to Lithium Disilicate Ceramic: Correlation between Flexural Strength and Modulus of Elasticity

**DOI:** 10.3390/polym15051128

**Published:** 2023-02-23

**Authors:** Masao Irie, Masahiro Okada, Yukinori Maruo, Goro Nishigawa, Takuya Matsumoto

**Affiliations:** 1 Department of Biomaterials, Okayama University Graduate School of Medicine, Dentistry and Pharmaceutical Science, 2-5-1 Shikata-cho, Okayama 700-8525, Japan; 2 Department of Prosthodontics, Division of Dentistry, Okayama University, 2-5-1 Shikata-cho, Okayama 700-8525, Japan

**Keywords:** shear bond strength, flexural strength, flexural modulus of elasticity, resin luting materials, durability, dual-cure vs. self-cure

## Abstract

This study investigates the effect of the curing mode (dual-cure vs. self-cure) of resin cements (four self-adhesive and seven conventional cements) on their flexural strength and flexural modulus of elasticity, alongside their shear bond strength to lithium disilicate ceramics (LDS). The study aims to determine the relationship between the bond strength and LDS, and the flexural strength and flexural modulus of elasticity of resin cements. Twelve conventional or adhesive and self-adhesive resin cements were tested. The manufacturer’s recommended pretreating agents were used where indicated. The shear bond strengths to LDS and the flexural strength and flexural modulus of elasticity of the cement were measured immediately after setting, after one day of storage in distilled water at 37 °C, and after 20,000 thermocycles (TC 20k). The relationship between the bond strength to LDS, flexural strength, and flexural modulus of elasticity of resin cements was investigated using a multiple linear regression analysis. For all resin cements, the shear bond strength, flexural strength, and flexural modulus of elasticity were lowest immediately after setting. A clear and significant difference between dual-curing and self-curing modes was observed in all resin cements immediately after setting, except for ResiCem EX. Regardless of the difference of the core-mode condition of all resin cements, flexural strengths were correlated with the LDS surface upon shear bond strengths (*R*^2^ = 0.24, *n* = 69, *p* < 0.001) and the flexural modulus of elasticity was correlated with them (*R*^2^ = 0.14, *n* = 69, *p* < 0.001). Multiple linear regression analyses revealed that the shear bond strength was 17.877 + 0.166, the flexural strength was 0.643, and the flexural modulus was (*R*^2^ = 0.51, *n* = 69, *p* < 0.001). The flexural strength or flexural modulus of elasticity may be used to predict the bond strength of resin cements to LDS.

## 1. Introduction

Esthetic resin cements are increasingly essential in dentistry for bonding and sealing of ceramics, indirect composite restorations, and CAD/CAM materials, owing to its excellent mechanical properties and tooth-colored or translucent characteristics. These cements traditionally require a bonding agent for adhesion to the tooth structure and primers for bonding to ceramic substrates [1,2,3,4,5,6]. Conventionally, resin cements are also categorized according to the following three polymerization modes and related applications, as the opacity and thickness of restorative materials do not allow for efficient photopolymerization: light-cured (e.g., cementation of laminate veneers), dual-cured (e.g., cementation of esthetical restorative materials) and self-cured (e.g., cementation of ceramic or metallic restoration, and posts or cores). Dual-cure resin cements have become very popular as they combine light and chemical curing methods. In a clinical setting, chemical polymerization (self-cured) is desired in cases where light irradiation is insufficient or unreachable. However, many reports have shown that the self-cure mode alone resulted in cements with lower mechanical properties compared with dual-cured resin cements. This tendency can be attributed to the degree of polymerization and consequent changes in mechanical properties (hardness, elastic moduli, and flexural strength) of resin cements. This is an important deficiency in resin cements with both conventional (non-adhesive) and self-adhesive types [3,4,5,6,7,8,9,10,11,12,13].

In recent years, a new type of adhesive bonding system categorized as “universal” has been introduced. Universal bonding is a one-step system that can be applied to enamel and dentin substrates in clinical situations. This universal bonding system can be applied to restorative materials, such as zirconia, metal, and various silicate-based ceramics without pre-treatment with priming agents [12,13,14,15,16].

All-ceramic restorations have also become popular due to an increase in the demand for aesthetic materials. Lithium disilicate ceramic (abbreviated as LDS) is a glass-ceramic that is compatible with either adhesive, self-adhesive, or conventional cementation, and is one of the most commonly used materials for single crowns owing to their esthetic optical properties [17].

The mechanical properties of restorative and luting materials have been evaluated using in vitro flexural testing [16,18,19,20,21,22,23]. In our recent paper, the shear bond strengths of luting agents to dentin and flexural moduli of elasticity were reported to increase after one day of storage. The relationship between flexural strength and the value of shear bond strength is more significant, as many fracture surfaces exhibit a cohesive failure mode [19,21,24,25]. As a result, it is very important to investigate the flexural properties (strength or modulus of elasticity) in detail in order to clarify the mechanisms of the shear bond strength, which could help to predict the success of bonding agents.

Therefore, this study sought to determine the effect of the cure mode of resin cement at three time points [after initial setting (i.e., immediate), after one day of storage in distilled water at 37 °C (i.e., one-day), and after 20,000 thermocycles (i.e., TC 20k)]. The null hypothesis was that the effect of curing mode and flexural properties of resin cements after TC 20k would influence the shear bond strength of resin cements to LDS. Their performance was assessed in terms of (a) flexural strength, (b) flexural modulus of elasticity, and (c) shear bond strength to LDS of dual-cured and self-cured resin cements. The hypothesis was that between the set of materials investigated, one or both properties of (a) and (b) would correlate with property (c).

## 2. Materials and Methods

### 2.1. Resin Cements and Self-Etch Adhesives

Details of 4 self-adhesive and 7 conventional (i.e., require a pre-treatment agent) resin cements and the manufacturer’s recommended pre-treating agents are summarized in Table 1 and Table 2. In this study, analysis of the shear bond strength to LDS was performed with conventional and self-adhesive resin cements as one group. The pretreating agent for LDS was also used for the self-adhesive type. This range of resin cement materials and adhesives systems was selected to represent the major restorative products currently used by dentists and also to provide comprehensive and clinically relevant results. A single operator (MI) performed all bonding procedures and mixing and handling were carried out according to the manufacturer’s recommendations (Table 2). Ten specimens were prepared for each material for evaluation of their mechanical properties at each time period (immediate, after one day, and after TC 20K). A visible light curing unit (New Light VL-II, GC, Tokyo, Japan; fiber optic tip diameter: 8 mm) was used to irradiate the light-activated materials for 20 s. Using a radiometer (Demetron/Kerr, Danbury, CT, USA), the light intensity was checked immediately before the application of each adhesive resin and composite filling material. During light curing, light intensity was maintained at 450 mW/cm^2^. Since the polymerization of Super-Bond Universal was in self-cure mode, all measurements were made only in self-cure mode. All procedures, except those for cavity preparation and mechanical testing, were performed in a thermo-hydrostatic room maintained at a temperature of 23 ± 0.5 °C and a relative humidity of 50 ± 2%.

### 2.2. Preparation of Lithium Disilicate Ceramics (LDS, IPS e.max CAD)

For each tested material, 90 custom-made blocks (LDS, IPS e.max CAD, Ivoclar Vivadent, Schaan, Liechtenstein; SiO_2_, Li_2_O, K_2_O, P_2_O_5_, ZrO_2_, ZnO, Al_2_O_3_, MgO, coloring oxides; diameter: 5 mm, thickness: 2 mm) were used and were embedded in a slow-setting epoxy resin (Epofix, Struers, Copenhagen, Denmark). Flat LDS surfaces were obtained by grinding with wet silicon carbide paper (# 600), pre-treated with a 4.5% hydrofluoric acid (HF, IPS ceramic etching gel, 20 s), as recommended by the manufacturer.

### 2.3. Measurement of Shear Bond Strengths to LDS

The resin-embedded LDS was fixed inside a mounting jig with a Teflon mold (diameter: 3.6 mm, height: 2.0 mm) set onto the LDS surface. The Teflon mold was then filled with each resin cement using a syringe tip (Centrix C-R Syringe System, Centrix, CT, USA), and a stainless-steel rod (diameter, 3.4 mm; height, 2 mm, pre-treated with air-abraded with 50 μm Al_2_O_3_ particles at 0.3 MPa pressure and Alloy Primer: Kuraray Noritake Dental) was placed onto each resin cement. Specimens were then submitted or not to light curing and evaluated at 3 time periods, as follows: dual-cure mode, D-(1) immediately after light activation, D-(2) after light curing and one day of storage in distilled water storage at 37 °C, and D-(3) after light curing and application of 20,000 thermocycles (thermal stress between 5 and 55 °C; 1 min dwell time; TC 20k); self-cure mode, S-(1) after initial setting and stored in 37 °C for 10 min, S-(2) after initial setting and stored in distilled water for one day at 37 °C, and S-(3) after initial setting and application of TC 20k. At each time period, a shear force was applied using a universal testing machine (Autograph AG-X 20kN, Shimadzu, Kyoto, Japan) at a crosshead speed of 0.5 mm/min. The force onto the stainless-steel rod was transmitted via a flat (blunt) 1-mm-thick shearing blade at a perpendicular direction to the load. Stress at failure was calculated and recorded as the shear bond strength. Failed specimens were examined under a light microscope at 4 magnifications (SMZ-10, Nikon, Tokyo, Japan) to determine the total number of adhesive failures [20,26,27,28].

### 2.4. Measurement of Flexural Strength and Flexural Modulus of Elasticity

Resin cement specimens for flexural testing (*n* = 10 per resin composite) were prepared in Teflon molds (25 × 2 × 2 mm^3^) and light-cured for 20 s or stored at 37 °C for 10 min for self-curing. Flexural strength was measured at 3 different time periods, as described in Section 2.3. Each cement specimen was subjected to a 3-point bending test with a 20-mm span and a load speed of 0.5 mm/min (Model 5565, Instron, Canton, MA, USA) as outlined in ISO 4049, and the flexural modulus was accordingly calculated using an accompanying software (Series IX software, Instron, Canton, MA, USA). All procedures, except for testing, were performed in an air-conditioned room at 23 ± 0.5 °C and 50 ± 2% R.H.

### 2.5. Statistical Analysis

Statistical analysis was performed using the software package Statistica 9.1 (Statsoft, OK, USA). Analysis of variance (2-way ANOVA) with Tukey-HSD for post hoc comparison was used to analyze the data obtained for the shear bond strength to LDS, flexural strength, and flexural modulus of elasticity. Comparison of the means for shear bond strength to LDS of each resin cement material with regard to 2 curing modes was analyzed by Student’s *t*-test. Any possible correlations between flexural strength and flexural modulus of elasticity were also evaluated by Spearman’s rank correlation coefficient. Analyses were conducted using SPSS version 19 (Chicago, IL, USA). Multiple linear regression analyses were conducted using the 3 independent factors: flexural strength, flexural modulus of elasticity, and shear bond strength. The level of significance was set at *p* < 0.05.

## 3. Results

### 3.1. Shear Bond Strength to LDS

The shear bond strengths of the resin cement materials and their statistical analyses are presented in Table 3, Table 4, Table 5 and Table 6. The shear bond strengths of all materials changed significantly with time. The higher mean values were observed after one day of storage or TC 20k (*p* < 0.05, Table 4). Although RelyX Universal Resin cement showed the greatest values among four conditions (two cure modes, after immediate setting, and one day of storage), Variolink Esthetic DC showed the greatest values in two conditions (two cure modes, after TC 20k). The cements were also statistically classified into groups as shown in Table 5. The most significant difference in the shear bond strength to LDS between self-cure and dual-cure modes was observed after immediate setting (*p* < 0.05). In contrast, after one day of storage and TC 20k, no significant differences in the effect of curing mode (i.e., self-cure and dual-cure) were observed on the shear bond strength to LDS (*p* > 0.05). There was no significant difference after TC 20k for all cements, except for RelyX Unicem 2 Automix and ResiCem EX (*p* > 0.05). In Table 6, the total number (pair) of statistical analyses (self-cure mode vs. dual-cure mode) was 33 (11 products multiplied by the three analyzed time periods). In summary, 10 pairs (30%) with significant differences and 23 pairs (70%) without significant differences were observed. Among the 10 pairs with significant differences, 7 of them showed a significant difference after initial setting. From this result, the most significant effect on the shear bond strength to LDS between pairs was re-confirmed to be after initial setting (immediate). In particular, the ResiCem EX pairs showed significant differences at all three time points (Table 6).

Regarding the failure mode, no adhesive fractures were observed (Table 3). In general, the proportion of adhesive failure modes was the same at all three time points.

### 3.2. Flexural Strength

The flexural strengths of the resin cement materials and their statistical analyses are presented in Table 7, Table 8, Table 9 and Table 10. The flexural strengths of all materials changed significantly with time. The higher mean values were observed after one day of storage or TC 20k (*p* < 0.05, Table 8). ESTECEM II showed the greatest values among all conditions, except for the self-cure mode after one day of storage, and RelyX Unicem 2 Automix showed the lowest values among half of the conditions. The cements were also statistically classified into groups as shown in Table 9. The significant differences in the flexural strength between self-cure and dual-cure modes (a pair) at each time point is shown in Table 10.

### 3.3. Flexural Modulus of Elasticity

The flexural modulus of elasticity of the resin cement materials and their statistical analyses are presented in Table 11, Table 12, Table 13 and Table 14. The flexural modulus of elasticity of all materials changed significantly with time. The higher mean values were seen after one day of storage or TC 20k (*p* < 0.05, Table 12).

ESTECEM II showed the greatest values among all conditions, while RelyX Universal Resin Cement showed the lowest values, except for self-curing mode after TC 20k. The cements were also statistically classified into groups as shown in Table 13. The significant differences in the flexural modulus of elasticity between self-cure and dual-cure modes (a pair) at each time point are shown in Table 14.

### 3.4. Correlation

**Figure 1:**
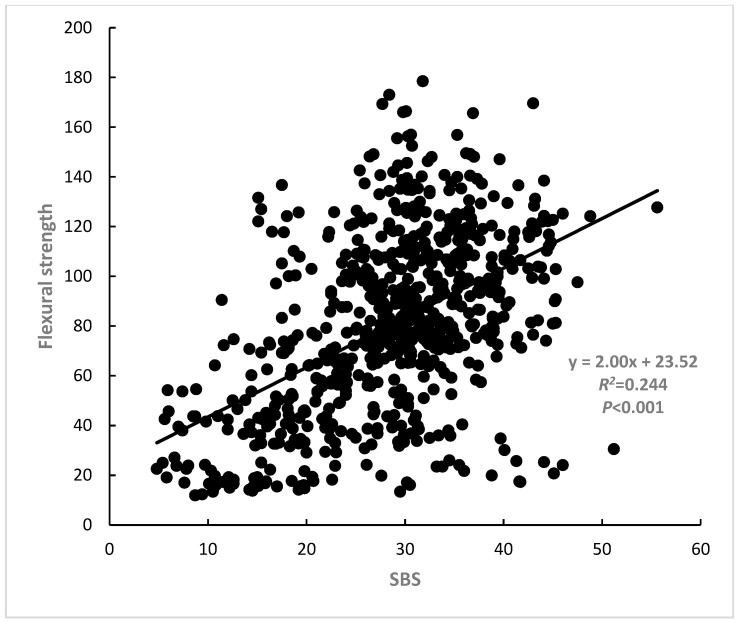
Correlation between flexural strength and shear bond strength to IPS e.max CAD (*R*^2^ = 0.24, *p* < 0.001).

**Figure 2:**
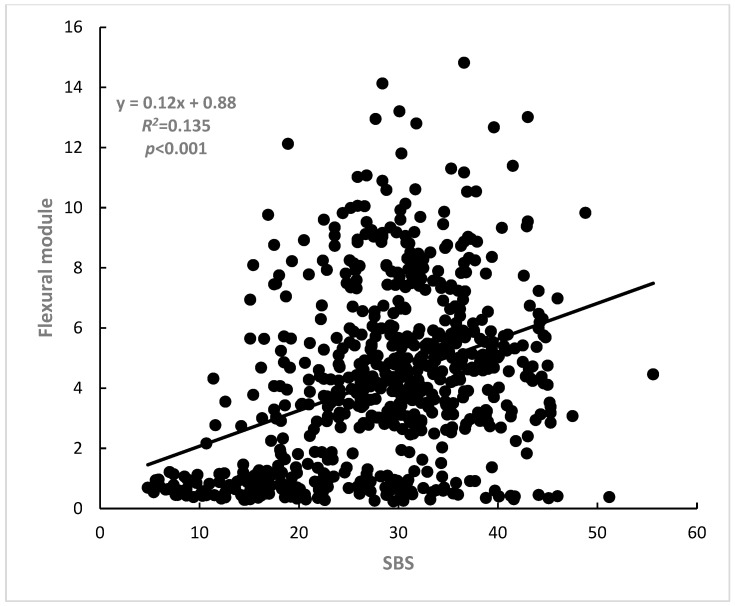
Correlation between flexural modulus of elasticity and shear bond strength to IPS e.max CAD (*R*^2^ = 0.14, *p* < 0.001).

**Figure 3:**
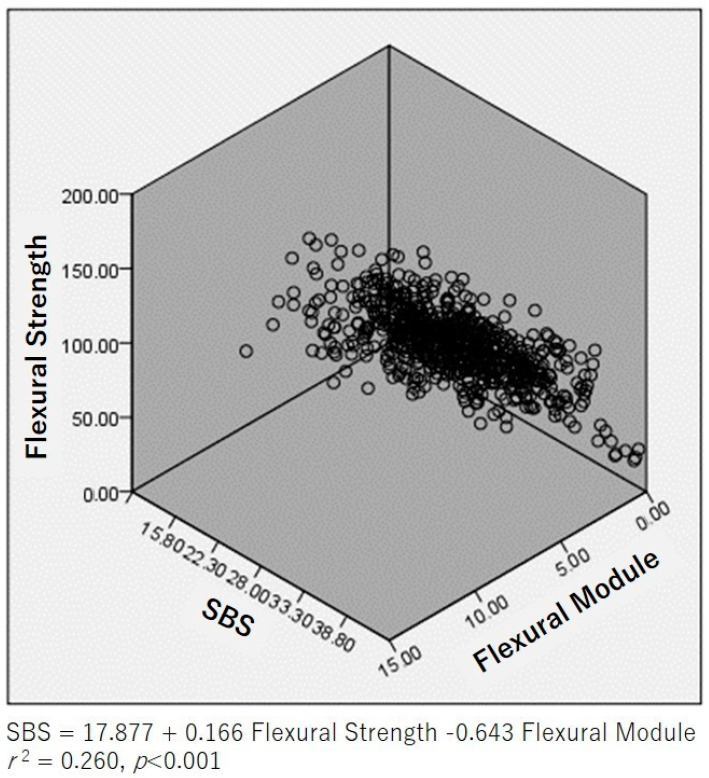
Correlation between shear bond strength to IPS e.max CAD, flexural strength, and flexural modulus of elasticity (*R*^2^ = 0.260, *p* < 0.001).

For all materials at the three time periods (dual-cure: *n* = 33, self-cure: *n* = 36), the relationships between the flexural strength and shear bond strength to LDS of both dual-curing and self-curing materials were analyzed and these relationships are presented as graphs in Figure 1, Figure 2 and Figure 3. Flexural strength was strongly correlated with shear bond strength to LDS (*R*^2^ = 0.24, *p* < 0.001). Flexural modulus of elasticity was strongly correlated with shear bond strength to LDS (*R*^2^= 0.14, *p* < 0.001). Multiple linear regression analyses were conducted using these three independent factors, and the following relationship was revealed: shear bond strength was 17.877 + 0.166, flexural strength was 0.643, and flexural modulus was *R*^2^ = 0.26, *n* = 69, *p* < 0.001). This section may be divided by subheadings. It should provide a concise and precise description of the experimental results, their interpretation, as well as the experimental conclusions that can be drawn.

## 4. Discussion

### 4.1. Shear Bond Strength to LDS

This study sought to unravel the effect of the curing mode of adhered resin cements to the LDS surface over three different times: immediately after curing, after one day of storage, and after TC 20k. The application of a silane-coupling agent is known to improve the bond strength to LDS and silica-based ceramics [9,10,29]. It was reported that chemical bonds between LDS and resin composite luting materials could be achieved by the silane group of silane molecules that react with silica on the ceramic surface [6,8,10,25,30]. Although all LDS surfaces in this study contained a silane-coupling agent, the results were dependent on the resin cement materials and analyzed time intervals [25].

### 4.2. Shear Bond Strength to LDS: After Immediate Setting to One Day of Storage

As a result of analysis with the combination of self-cure vs. dual-cure modes, a significant difference was found in 70% of the cases after initial setting (immediate) (*p* < 0.05). In other words, it was clear that the value of the shear bond strength in the self-cure mode was significantly lower in seven pair groups. At first, as can be seen from the results of the flexural strength analysis (Table 7), the difference in the polymerization rate is considered to be large [3,4,9]. Moreover, since the surface of LDS retreats with 4.5% HF solution, a large number of micro-mechanical retentions could be formed [11]. Therefore, it seems that the difference in the initial polymerization rate affected the reaction at the interface between the luting material and LDS [9] and the micromechanical inter-locking force [23]. From these results, the null hypothesis could be accepted.

Although the polymerization rate on the surfaces of the materials after initial setting (i.e., the dual-curing and self-curing modes) were estimated using attenuated total reflection Fourier-transform infrared (ATR FT-IR) spectroscopy measurements, the expected results were not obtained (data not shown).

### 4.3. Shear Bond Strength to LDS: After One Day of Storage to TC 20k

As a result of the analysis with the combination of self-curing vs. dual-curing modes, no significant difference was seen, except for RelyX Unicem 2 Automix and ResiCem EX (Table 6). Contrary to the results observed after immediate setting to one day of storage, there were no significant differences in the shear bond strength to LDS after one day of storage to TC 20k. A major reason could be related to the similar polymerization rate after TC 20k independent of the curing mode. A water-soluble chemical polymerization catalyst was introduced to enhance the polymerization performance even in the presence of water. It is thought that the combination, ratio of co-monomers, a new functional unique monomer, and the silanized effect provided the ability to create a strong bond to the LDS surface and good storage stability (Table 2). As a result of the adhesive effectiveness of a pre-activated silane solution based on gamma-MPTS, there was no significant difference in cement bond strength between 15-min storage and 1-month storage (the same immersion time as TC 20k) [30]. By analogy, it is considered that the result was not significantly different. From this result from one-day storage to TC 20k, the null hypothesis could be rejected.

Importantly, decreased flexural strength in the many resin cements was observed after TC 20k. According to the polymer engineering theory, there are three mechanisms of composite water uptake: diffusion of water molecules within the matrix, infiltration at the matrix–filler interface, and absorption into the microcracks produced by incubation in high and low temperatures. Water uptake causes the matrix to expand which induces stress inside the material. However, such a decrease in flexural modulus was not evident. Furthermore, long-term immersion in water may not have had such a severe effect (Table 12).

### 4.4. Relation to Flexural Strength and Flexural Modulus of Elasticity

The type of bond strength test was categorized in terms of the mechanical loading direction. Almost all bond strength testing was categorized as shear or tensile bond strength. Flexural strength testing is sensitive to surface defects, such as cracks, voids, and scratches, which can influence the fracture characteristics of a brittle material. The flexural strength and flexural modulus of elasticity are very important for testing shear bond strength. The degree of high flexural strength and flexural modulus of elasticity is believed to reflect the high resistance to surface defects and erosion. Therefore, the flexural strength and flexural modulus of elasticity are thought to be significant mechanical properties of luting materials [14,19,20,21,23,25,31], unlike compressive strength and tensile strength. To improve the mechanical properties of these luting materials, development and relation efforts should focus on the change in flexural properties over time [14,23]. As the result, this investigation was carried out with luting materials at three different time periods to evaluate their flexural property performance in relation to their shear bond strength to LDS, by also evaluating the effect of both dual-curing and self-curing modes.

Most of the fracture modes observed after shear bond testing were mixed and cohesive failures were also observed, which are in agreement with previous studies [14,21,25,31]. From this fracture mode, it is considered that the flexural property of the luting material itself has a great effect on the shear bond strength. Therefore, it is conceivable that the bond strength to LDS improved as both the flexural strength and flexural modulus of elasticity of cement itself increased. As the results show, the bond strength of resin cements to LDS was correlated with their flexural strength (*R*^2^ = 0.244, *p* < 0.001, integrating self-cure and dual-cure), as well as with their flexural modulus of elasticity (*R*^2^ = 0.135, *p* < 0.001, integrating self-cure and dual-cure).

Multiple linear regression analyses were conducted using the three independent factors of shear bond strength to LDS, flexural strength, and flexural modulus of elasticity. They revealed this relationship: shear bond strength = 17.877 + 0.166, a flexural strength of 0.643, and a flexural modulus of elasticity of *R*^2^ = 0.26, *n* = 69, *p* < 0.001). In other words, regardless of the luting material, the shear bond strength to LDS was correlated to flexural strength and flexural modulus of elasticity.

The vectors of loading for shear bond strength and in cases such as three-point bending strength are similar. In particular, when measuring the three-point bending strength, shear loads may be applied to both ends of the cylinder, which is indicated by the cylinder, and these two characteristics are considered to have a high relationship.

From these results, it was shown that not only flexural strength, but also flexural modulus of elasticity, is significantly related to shear bond strength to LDS.

### 4.5. Limitations

The limitations of the present study are inherent to the in vitro design, where only controlled variables are considered. Intra-oral temperature changes may influence the long-term outcome of indirect restoration, since the different materials employed in the study present higher thermal contraction/expansion coefficients than teeth [3].

## 5. Conclusions

1. As a result of the analysis of 33 combinations of self-cure vs. dual-cure mode regarding shear bond strength to LDS, no significant difference was shown in 70% of the analyzed resin cements.
2. Multiple linear regression analyses using shear bond strength to LDS, flexural strength, and flexural modulus of elasticity as independent factors, showed this relationship: a shear bond strength of 17.877 + 0.166, a flexural strength of 0.643, and a flexural modulus of *R*^2^ = 0.260, *n* = 69, *p* < 0.001).

## Figures and Tables

**Table 1:** Luting agent materials investigated.

Product	Composition	Manufacturer	Batch No.
RelyX Universal Resin Cement	Surface-treated glass powder filler, phosphate ester monomer, TEGDMA, diurethane dimethacrylate, silica filler, initiator, titanium dioxide.	3M, Seefeld, Germany	7304443
RelyX Ultimate	Surface-treated glass powder filler, phosphate ester monomer, TEGDMA, 1,12-dodecane dimethaycrylate, silica filler, initiator, calcium hydroxide, titanium dioxide, filler content: 43 vol%, 70 wt.%,	3M, Seefeld, Germany	6798079
RelyX Unicem 2 Automix	Surface-treated glass powder filler, phosphate ester monomer, TEGDMA, 1,12-dodecane dimethaycrylate, silica filler, initiator, calcium hydroxide, sodium p-toluensulfinatet, methacrylated amine, titanium dioxide, filler content: 43 vol%, 70 wt.%.	3M, Seefeld, Germany	6485786
PANAVIA V5	Paste A: bis-GMA, TEGDMA, hydrophobic aromatic dimethacrylate, hydrophilic aliphatic dimethacrylate, initiators, accelerators, silanated barium glass filler, silanated fluoroalminosilicate glass filler, colloidal silica. Paste B: bis-GMA, hydrophobic aromatic dimethacrylate, hydrophilic aliphatic dimethacrylate, silanated barium glass filler, silanated alminium oxide filler, accelerators, dl-camphorquinone, pigments, filler content: 38 vol%, 61 wt.%.	Kuraray Noritake Dental, Tainai, Niigata, Japan	2Q0128
PANAVIA SA Cement Universal (Automix)	Paste A: MDP, bis-GMA, TEGDMA, HEMA, silanated barium glass filler, silanated colloidal silica, dl-camphorquinone, peroxide, catalysts, pigments. Paste B: hydrophobic aromatic dimethacrylate, silane coupling agent, silanated barium glass filler, aluminum oxide filler, surface-treated sodium fluoride (less than 1%), dl-camphorquinone, accelerators, pigments, filler content: 40 vol%, 62 wt.%.	Kuraray Noritake Dental, Tainai, Niigata, Japan	BL0037
G-Cem ONE	Fluoro-alumino-silicate-glass, dimethacrylate, phosphoric ester monomer, silicone dioxide, initiator.	GC Corporation,Tokyo, Japan	2012171
ESTECEM II	Paste A: bis-GMA, TEGDMA, bis-MPEPP, silica–zirconia filler. Paste B: bis-GMA, TEGDMA, bis-MPEPP, silica–zirconia filler, camphorquinone, peroxide, filler content: 74 wt.%.	Tokuyama Dental, Tokyo, Japan	078001
SpeedCEM Plus	Monomer matrix: dimethacrylates and acidic monomers. Inorganic filler: barium glass, ytterbium trifluoride, co-polymerand highly dispersed silicon dioxide. Additional contents: initiators, stabilizers, color pigments (<1%). Inorganic fillers: 0.1–7 micro-meter, total content of inorganic filler: approx. 70 wt.%.	Ivoclar Vivadent AG, Schaan, Liechtenstein	ZOOMRJ
Variolink Esthetic DC	Monomer matrix: urethane dimethacrylate and further methacrylate monomers. Inorganic filler: mixed oxides, ytterbium trifluoride. Additional contents: initiators, stabilizers, pigments (<1%). Total content of inorganic filler: approx. 65 wt.%.	Ivoclar Vivadent AG, Schaan, Liechtenstein	ZO1FPV
ResiCem EX	Paste A: S-PRG filler, bis-GMA, silicate glass, initiators, others. Paste B: S-PRG filler, bis-GMA, silicate glass, initiators, others. Filler content: 65 wt.%, 45 vol.%.	Shofu, Kyoto, Japan	062103
Nexus Universal Chroma	TEGDMA, urethane dimethacrylate, bisphenol A diglycidylmethacrylate (bis-GMA), GPDM, photoinitiator, redox initiator, bariumaluminosilicate glass filler, ytterbium fluoride, silica. Filler content: 43 vol.%, 68 wt.%.	Kerr, Orange CA, USA	8224016
Super-Bond Universal	PMMA, 4-META, MMA, TBB, self-cure type (bulk-mix technique).	SUN MEDICAL, Moriyama, Japan	Universal Polymer: TW1, Quick Monomer: VK1, Catalyst V: TT22

EGDMA, triethyleneglycol dimethacrylate; bis-GMA, bisphenol A diglycidylmethacrylate; MDP, 10-methacryloyloxydecyl dihydrogen phosphate; HEMA, 2-hydroxymethacrylate; PMMA, poly(methyl methacrylate); 4-META, 4-methacryloxyethyl trimellitate anhydride; MMA, methyl methacrylate; TBB, tri-n-butylborane.

**Table 2:** Self-etching adhesives and system adhesive components.

Adhesive	Batch No.	Composition	Manufacturer	Surface Treatment of IPS e.max
Scotchbond Universal Plus Adhesive	6840217	Brominated dimethacrylate, HEMA, silane-treated silica, Vitrabond copolymer, MDP, initiators, silane, ethanol.	3M, Seefeld, Germany	Scotchbond Universal Plus Adhesive (20 s)–air (5 s)
Scotchbond Universal Adhesive	596935	bis-GMA, HEMA, decamethylene dimethacrylate, silane-treated silica, Vitrabond copolymer, MDP, initiators, silane, ethanol.	3M, Seefeld, Germany	Scotchbond Universal Adhesive (20 s)–air (5 s)
RelyX Ceramic Primer	NC25837	Water, thyl alcohol, 3-methacryloxyoripyl trimethoxysilane.	3M, Seefeld, Germany	RelyX Ceramic Primer–air (5 s)
CLEAFIL CERAMIC PRIMER PLUS	2R0052	3-methacryloxypropyl trimethoxysilane, MDP, ethanol.	Kuraray Noritake Dental, Tainai, Niigata, Japan	CLEAFIL CERAMIC PRIMER PLUS (1–2 s)–air (5 s)
G-Multi PRIMER	2104221	Vinyl silane, phosphate ester monomer, thiophosphate ester monomer,methacrylic ester, ethanol	GC Corporation,Tokyo, Japan	G-Multi PRIMER application—air dry
BONDMER lightless II	Liquid A: KA200731 Liquid B: KB200702	Liquid A: phosphoric acid monomer (new 3D-SR monomer), MTU-6, HEMA, bis-GMA, TEGDMA, acetone, others. Liquid B: γ-MPTES, borate, peroxide, acetone, ethanol, water, others.	Tokuyama Dental, Tokyo, Japan	BONDMER lightless II (1-2 s)–air (5 s)
Monobond Plus	ZO1LG8	Phosphoric acid monomer, silane methacrylate, ethanol.	Ivoclar Vivadent AG, Schaan, Liechtenstein	Monobond Plus (60 s)–air
BeautiBond Xtreme	112003	Acetone, water, bis-GMA, TEGDMA, phosphoric ester monomer, silane coupling agent, initiator, others.	Shofu, Kyoto, Japan	BeautiBond Xtreme (20 s)–air
OptiBond eXTRa Universal	Adhesive: 8181793	HEMA, dimethacrylate monomers, tri-functional methacrylate monomer, ethanol, photo-initiator, bariumaluminosilicate filler, silica, sodium hexafluorosilicate.	Kerr, Orange CA, USA	OptiBond eXTRa Adhesive (15 s)–air (5 s)–LED light (5 s)
M&C Primer A and B	Liquid A: TV1, Liquid B: TV11	M&C Primer A: MDP, VTD, MMA. Acetone M&C Primer B: γ-MPTS, MMA.	SUN MEDICAL, Moriyama, Japan	Mix (Liquid A + Liquid B,1–2 s)–air (5 s)
Scotchbond Universal Plus Adhesive	6840217	Brominated dimethacrylate, HEMA, silane-treated silica, Vitrabond copolymer, MDP, initiators, silane, ethanol.	3M, Seefeld, Germany	8224016
Scotchbond Universal Adhesive	596935	Bis-GMA, HEMA, decamethylene dimethacrylate, silane-treated silica, Vitrabond copolymer, MDP, initiators, silane, ethanol.	3M, Seefeld, Germany	Universal Polymer: TS1, Catalyst V: TT22

2-HEMA, hydroxyethylmethacrylate; MDP, 10-methacryloyloxydecyl dihydrogen phos-phate; Bis-GMA, bisphenol A diglycidylmethacrylate; 4-MET, 4-methacryloxyethyl trimellitic acid; MTU-6, 6-methacryloxyhexyl 2-thiouracil-5-carboxylate; γ-MPTES, 3-(triethoxysilyl) propyl methacrylate; VTD, 6-(4-vinylbenzyl-n-propyl) amino-1,3,5-triazine-2,4-dithione; MMA, methyl methacrylate; γ-MPTS, 3-methacryloxypropyl trimethoxy silane.

**Table 3:** Shear bond strength to LDS (e.max) of the adhesive resin cements (MPa, mean (S.D.), Adh.).

Resin Cement/Pretreating Agent	Cure Mode	Time		
		Immediate	After One-Day Storage	TC 20k
RelyX Universal Resin Cement/ Scotchbond Universal Plus Adhesive	Dual	32.5 (5.0, 0)	43.4 (5.1, 0)	29.3 (3.3, 0)
	Self	28.7 (4.6, 0)	42.5 (4.3, 0)	27.1 (4.2, 0)
RelyX Ultimate/Scotchbond Universal Adhesive	Dual	31.8 (3.1, 0)	35.5 (6.2, 0)	22.3 (3.6, 0)
	Self	14.7 (3.3, 0)	33.2 (4.0, 0)	24.4 (4.4, 0)
RelyX Unicem 2 Automix/ RelyX Ceramic Primer	Dual	21.1 (4.0, 0)	33.0 (4.9, 0)	28.6 (2.8, 0)
	Self	12.1 (2.7, 0)	36.0 (5.8, 0)	33.4 (5.5, 0)
PANAVIA V5/ Clearfil Ceramic Primer Plus	Dual	26.4 (5.9, 0)	30.9 (4.0, 0)	30.8 (3.7, 0)
	Self	23.6 (6.6, 0)	33.4 (5.8, 0)	32.4 (4.0, 0)
PANAVIA SA Cement Universal/None	Dual	12.2 (3.9, 0)	38.3 (3.7, 0)	33.7 (6.6, 0)
	Self	15.1 (3.7, 0)	40.8 (5.7, 0)	29.2 (5.2, 0)
G-Cem ONE/G-Multi PRIMER	Dual	19.0 (3.8, 0)	31.2 (4.6, 0)	26.2 (3.4, 0)
	Self	18.3 (3.0, 0)	30.8 (3.7, 0)	28.2 (4.9, 0)
ESTECEM II/ BONDMER Lightless II	Dual	25.2 (4.8, 0)	33.3 (6.0, 0)	31.1 (4.9, 0)
	Self	20.2 (4.3, 0)	29.9 (5.7, 0)	30.2 (2.3, 0)
SpeedCEM Plus/ Monobond Plus	Dual	21.7 (3.7, 0)	35.4 (5.2, 0)	33.8 (3.1, 0)
	Self	7.3 (1.8, 0)	35.5 (4.0, 0)	31.2 (3.4, 0)
Variolink Esthetic DC/ Monobond Plus	Dual	30.2 (3.8, 0)	41.6 (3.7, 0)	35.8 (5.5, 0)
	Self	18.2 (2.7, 0)	39.1 (5.8, 0)	35.3 (4.4, 0)
ResiCem EX/ BeautiBond Xtreme	Dual	14.8 (3.1, 0)	31.2 (3.3, 0)	30.5 (3.3, 0)
	Self	9.3 (3.2, 0)	27.6 (2.5, 0)	23.9 (3.5, 0)
Nexus Universal Chroma/ +OptiBond eXTRa Universal	Dual	20.9 (2.9, 0)	37.1 (6.1, 0)	31.8 (4.6, 0)
	Self	16.9 (2.6, 0)	34.1 (4.4, 0)	29.2 (3.6, 0)
Super-Bond Universal/M&C Primers A and B	Dual	----	----	----
	Self	4.8 (1.2, 0)	33.2 (3.8, 0)	30.9 (1.6, 0)

*n* = 10, Adh: number of adhesive failure modes after failure 5, TC 20k: after 20,000 thermocycles.

**Table 4:** Comparison of the means (Tukey HSD Procedure) for shear bond strength to LDS (e.max) of each adhesive resin cement material with regard to time [superscript letters (a–g) represent groups with no significant difference, *p* > 0.05].

RelyX Universal Resin Cement/Scotchbond Universal Plus Adhesive		RelyX Ultimate/Scotchbond Universal Plus Adhesive		RelyX Unicem 2 Automix/RelyX Ceramic Primer		PANAVIA V5/Clearfil Ceramic Primer Plus	
Dual-cure mode	Self-cure mode	Dual-cure mode	Self-cure mode	Dual-cure mode	Self-cure mode	Dual-cure mode	Self-cure mode
TC 20k ^a^	TC 20k ^b^	TC 20k	Immediate	Immediate	Immediate	Immediate ^f^	Immediate
Immediate ^a^	Immediate ^b^	Immediate ^c^	TC 20k	TC 20k ^d^	One-day ^e^	TC 20k ^f^	TC 20k ^g^
One-day	One-day	One-day ^c^	One-day	One-day ^d^	TC 20k ^e^	One-day ^f^	One-day ^g^
PANAVIA SA Cement Universal/None		G-Cem ONE/G-Multi PRIMER		ESTECEM II/BONDMER Lightless II		SpeedCEM Plus/Monobond Plus	
Dual-cure mode	Self-cure mode	Dual-cure mode	Self-cure mode	Dual-cure mode	Self-cure mode	Dual-cure mode	Self-cure mode
TC 20k ^a^	TC 20k ^b^	TC 20k	Immediate	Immediate	Immediate	Immediate ^f^	Immediate
Immediate ^a^	Immediate ^b^	Immediate ^c^	TC 20k	TC 20k ^d^	One-day ^e^	TC 20k ^f^	TC 20k ^g^
One-day	One-day	One-day ^c^	One-day	One-day ^d^	TC 20k ^e^	One-day ^f^	One-day ^g^
Variolink Esthetic DC/Monobond Plus		ResiCem EX/BeautiBond Xtreme		Nexus Universal Chroma/OptiBond eXTRa Universal		Super-Bond Universal/M&C Primers A and B	
Dual-cure mode	Self-cure mode	Dual-cure mode	Self-cure mode	Dual-cure mode	Self-cure mode	Dual-cure mode	Self-cure mode
TC 20k ^a^	TC 20k ^b^	TC 20k	Immediate	Immediate	Immediate	-	Immediate
Immediate ^a^	Immediate ^b^	Immediate ^c^	TC 20k	TC 20k ^d^	One-day ^e^	-	TC 20k ^g^
One-day	One-day	One-day ^c^	One-day	One-day ^d^	TC 20k ^e^	-	One-day ^g^

*n* = 10, Adh: Number of adhesive failure modes after failure 5), TC 20k: after 20,000 thermocycles.

**Table 5:** Comparison of means (Tukey HSD procedure) for shear bond strength of adhesive resin cement material * at each time period [superscript letters (a–i) represent groups with no significant difference, *p* > 0.05].

Immediate		After One-Day Storage		After TC 20k	
Dual-Cure Mode	Self-Cure Mode	Dual-Cure Mode	Self-Cure Mode	Dual-Cure Mode	Self-Cure Mode
PANAVIA SA Cement Universal ^a^	Super-Bond Universal ^a^	PANAVIA V5 ^a^	ResiCem EX ^a^	RelyX Ultimate ^a^	ResiCem EX ^a^
ResiCem EX ^a b^	SpeedCEM Plus ^a b^	ResiCem EX ^a^	G-Cem ONE ^a b^	G-Cem ONE ^a b^	RelyX Ultimate ^a^
ESTECEM II ^a b c^	ResiCem EX ^a b c^	G-Cem ONE^a b^	RelyX Ultimate ^a b c^	RelyX Unicem 2 Automix ^a b c^	RelyX Universal Resin Cement ^a b^
G-Cem ONE ^b c^	RelyX Unicem 2 Automix ^b c d^	ESTECEM II ^a b^	Super-Bond Universal ^a b c^	RelyX Universal Resin Cement ^b c^	G-Cem ONE ^a b c^
Nexus Universal Chroma ^c d^	RelyX Ultimate ^c d e^	RelyX Unicem 2 Automix ^a b^	PANAVIA V5 ^a b c^	ResiCem EX ^b c d^	PANAVIA SA Cement Universal ^a b c^
RelyX Unicem 2 Automix ^c d^	PANAVIA SA Cement Universal ^d e f^	SpeedCEM Plus ^a b c^	ESTECEM II ^a b c d^	PANAVIA V5 ^b c d^	Nexus Universal Chroma ^a b c^
SpeedCEM Plus ^c d^	Nexus Universal Chroma ^d e f^	RelyX Ultimate ^a b c^	Nexus Universal Chroma ^a b c d^	Nexus Universal Chroma^b c d^	Super-Bond Universal ^b c^
PANAVIA V5 ^d e^	G-Cem ONE ^e f g^	Nexus Universal Chroma ^a b c d^	SpeedCEM Plus ^b c d^	ESTECEM II ^c d^	SpeedCEM Plus ^b c^
Variolink Esthetic DC ^e f^	ESTECEM II ^f g^	PANAVIA SA Cement Universal ^b c d^	RelyX Unicem 2 Automix ^b c d e^	PANAVIA SA Cement Universal ^c d^	PANAVIA V5 ^b c^
RelyX Ultimate ^e f^	PANAVIA V5 ^g h^	Variolink Esthetic DC ^c d^	Variolink Esthetic DC ^c d e^	SpeedCEM Plus ^c d^	ESTECEM II ^b c^
RelyX Universal Resin Cement ^f^	RelyX Universal Resin Cement ^h i^	RelyX Universal Resin Cement ^d^	PANAVIA SA Cement Universal ^d e^	Variolink Esthetic DC ^d^	RelyX Unicem 2 Automix ^c^
	Variolink Esthetic DC ^i^		RelyX Universal Resin Cement ^e^		Variolink Esthetic DC ^c^

TC 20k: after 20,000 thermocycles, * pre-treated primers were deleted.

**Table 6:** Comparison of the results (by *t*-test) for shear bond strength to LDS (e.max) of each adhesive resin cement material with regard to two curing modes.

RelyX Universal Resin Cement/Scotchbond Universal Plus Adhesive			RelyX Ultimate/Scotchbond Universal Plus Adhesive			RelyX Unicem 2 Automix/RelyX Ceramic Primer			PANAVIA V5/Clearfil Ceramic Primer Plus		
Immediate	One-day	TC 20k	Immediate	One-day	TC 20k	Immediate	One-day	TC 20k	Immediate	One-day	TC 20k
NS	NS	NS	S	NS	NS	S	NS	S	NS	NS	NS
PANAVIA SA Cement Universal/None			G-Cem ONE/ G-Multi PRIMER			ESTECEM II/ BONDMER Lightless II			SpeedCEM Plus/ Monobond Plus		
Immediate	One-day	TC 20k	Immediate	One-day	TC 20k	Immediate	One-day	TC 20k	Immediate	One-day	TC 20k
NS	NS	NS	NS	NS	NS	S	NS	NS	S	NS	NS
Variolink Esthetic DC/ Monobond Plus			ResiCem EX/ BeautiBond Xtreme			Nexus Universal Chroma/ OptiBond eXTRa Universal					
Immediate	One-day	TC 20k	Immediate	One-day	TC 20k	Immediate	One-day	TC 20k			
S	NS	NS	S	S	S	S	NS	NS			

*n* = 10, Adh: Number of adhesive failure modes after failure 5, TC 20k: after 20,000 thermocycles.

**Table 7:** Flexural strength of resin cements [MPa, mean (S.D.)].

Resin Cement	Cure Mode	Time		
		Immediate	After One-Day Storage	TC 20k
RelyX Universal Resin Cement	Dual	65.6 (0.3)	117.5 (7.0)	108.6 (8.8)
	Self	18.8 (3.5)	76.0 (3.9)	68.6 (5.5)
RelyX Ultimate	Dual	71.4 (4.6)	119.4 (4.6)	100.3 (4.9)
	Self	15.6 (1.3)	82.8 (6.9)	68.0 (4.3)
RelyX Unicem 2 Automix	Dual	71.9 (5.7)	108.0 (6.8)	83.8 (5.1)
	Self	16.1 (2.8)	78.7 (7.0)	76.0 (7.8)
PANAVIA V5	Dual	35.8 (3.6)	144.8 (8.2)	121.9 (10.1)
	Self	38.5 (4.2)	95.1 (6.0)	93.6 (4.9)
PANAVIA SA Cement Universal (Automix)	Dual	38.3 (3.6)	109.3 (5.2)	111.6 (9.8)
	Self	17.2 (1.7)	78.4 (6.8)	84.9 (8.9)
G-Cem ONE	Dual	44.6 (3.1)	132.4 (7.1)	106.9 (6.0)
	Self	27.1 (5.8)	92.8 (11.8)	80.8 (7.4)
ESTECEM II	Dual	123.1 (7.7)	162.1 (11.7)	146.4 (10.1)
	Self	50.5 (8.7)	94.4 (8.3)	94.0 (8.4)
SpeedCEM Plus	Dual	55.2 (8.0)	107.7 (7.5)	91.4 (8.3)
	Self	22.7 (2.9)	79.4 (5.8)	59.1 (6.4)
Variolink Esthet DC	Dual	38.2 (3.7)	123.4 (5.7)	104.2 (5.8)
	Self	38.1 (6.5)	94.5 (5.8)	56.8 (7.7)
ResiCem EX	Dual	75.8 (8.3)	133.5 (6.2)	122.4 (9.1)
	Self	47.9 (6.0)	91.1 (10.7)	60.3 (5.1)
Nexus Universal Chroma	Dual	42.6 (4.8)	133.3 (9.2)	114.2 (8.6)
	Self	37.3 (7.1)	75.5 (8.5)	58.3 (6.6)
Super-Bond Universal	Dual	NA	NA	NA
	Self	23.5 (4.7)	100.6 (7.1)	79.4 (6.6)

*n* = 10, TC 20k: after 20,000 thermocycles.

**Table 8:** Comparison of the means (Tukey HSD procedure) for flexural strength of each resin cement material with regard to time [superscript letters (a–g) represent groups with no significant difference, *p* > 0.05].

RelyX Universal Resin Cement		RelyX Ultimate		RelyX Unicem 2 Automix		PANAVIA V5	
Dual-cure mode	Self-cure mode	Dual-cure mode	Self-cure mode	Dual-cure mode	Self-cure mode	Dual-cure mode	Self-cure mode
TC 20k ^a^	TC 20k ^b^	TC 20k	Immediate	Immediate	Immediate	Immediate ^f^	Immediate
Immediate ^a^	Immediate ^b^	Immediate ^c^	TC 20k	TC 20k ^d^	One-day ^e^	TC 20k ^f^	TC 20k ^g^
One-day	One-day	One-day ^c^	One-day	One-day ^d^	TC 20k ^e^	One-day ^f^	One-day ^g^
PANAVIA SA Cement Universal		G-Cem ONE		ESTECEM II		SpeedCEM Plus	
Dual-cure mode	Self-cure mode	Dual-cure mode	Self-cure mode	Dual-cure mode	Self-cure mode	Dual-cure mode	Self-cure mode
TC 20k ^a^	TC 20k ^b^	TC 20k	Immediate	Immediate	Immediate	Immediate ^f^	Immediate
Immediate ^a^	Immediate ^b^	Immediate ^c^	TC 20k	TC 20k ^d^	One-day ^e^	TC 20k ^f^	TC 20k ^g^
One-day	One-day	One-day ^c^	One-day	One-day ^d^	TC 20k ^e^	One-day ^f^	One-day ^g^
Variolink Esthetic DC		ResiCem EX		Nexus Universal Chroma		Super-Bond Universal	
Dual-cure mode	Self-cure mode	Dual-cure mode	Self-cure mode	Dual-cure mode	Self-cure mode	Dual-cure mode	Self-cure mode
TC 20k ^a^	TC 20k ^b^	TC 20k	Immediate	Immediate	Immediate	-	Immediate
Immediate ^a^	Immediate ^b^	Immediate ^c^	TC 20k	TC 20k ^d^	One-day ^e^	-	TC 20k ^g^
One-day	One-day	One-day ^c^	One-day	One-day ^d^	TC 20k ^e^	-	One-day ^g^

TC 20k: after 20,000 thermocycles.

**Table 9:** Comparison of means (Tukey HSD procedure) for flexural strength of resin cement material at each time [superscript letters (a–f) represent groups with no significant difference, *p* > 0.05].

Immediate		After One-Day Storage		After TC 20k	
Dual-Cure Mode	Self-Cure Mode	Dual-Cure Mode	Self-Cure Mode	Dual-Cure Mode	Self-Cure Mode
PANAVIA V5 ^a^	RelyX Ultimate ^a^	RelyX Unicem 2 Automix ^a^	Nexus Universal Chroma ^a^	RelyX Unicem 2 Automix ^a^	Variolink Esthetic DC ^a^
Variolink Esthetic DC ^a b^	RelyX Unicem 2 Automix ^a b^	PANAVIA SA Cement Universal ^a b^	RelyX Universal Resin Cement ^a^	SpeedCEM Plus ^a b^	Nexus Universal Chroma ^a b^
PANAVIA SA Cement Universal ^a b^	PANAVIA SA Cement Universal ^a b^	SpeedCEM Plus ^a b c^	PANAVIA SA Cement Universal ^a^	RelyX Ultimate ^b c^	SpeedCEM Plus ^a b c^
Nexus Universal Chroma ^a b^	RelyX Universal Resin Cement ^a b^	RelyX Universal Resin Cement ^a b c^	RelyX Unicem 2 Automix ^a^	Variolink Esthetic DC ^b c d^	ResiCem EX ^a b c^
G-Cem ONE ^b^	SpeedCEM Plus ^a b c^		SpeedCEM Plus ^a^	G-Cem ONE ^b c d^	RelyX Ultimate ^b c d^
SpeedCEM Plus ^c^	Super-Bond Universal ^b c^	Variolink Esthetic DC^c d^	RelyX Ultimate ^a b^	RelyX Universal Resin Cement ^c d^	RelyX Universal Resin Cement ^c d^
RelyX Universal Resin Cement ^d^	G-Cem ONE ^c^	G-Cem ONE ^d^	ResiCem EX ^b c^	PANAVIA SA Cement Universal ^c d e^	RelyX Unicem 2 Automix ^d e^
RelyX Ultimate ^d e^	Nexus Universal Chroma ^d^	Nexus Universal Chroma ^d^	G-Cem ONE ^b c^	Nexus Universal Chroma^d e^	Super-Bond Universal ^e^
RelyX Unicem 2 Automix ^d e^	Variolink Esthetic DC ^d^	ResiCem EX ^d^	ESTECEM II ^c^	PANAVIA V5 ^e^	G-Cem ONE ^e^
ResiCem EX ^e^	PANAVIA V5 ^d^	PANAVIA V5 ^e^	Variolink Esthetic DC ^c^	ResiCem EX ^e^	PANAVIA SA Cement Universal ^e f^
ESTECEM II ^f^	ResiCem EX ^e^	ESTECEM II ^f^	PANAVIA V5 ^c^	ESTECEM II ^f^	PANAVIA V5 ^f^
	ESTECEM II ^e^		Super-Bond Universal^c^		ESTECEM II ^f^

TC 20k: after 20,000 thermocycles.

**Table 10:** Comparison of the results (by *t*-test) for flexural strength of each adhesive resin cement material with regard to two curing modes.

RelyX Universal Resin Cement			RelyX Ultimate			RelyX Unicem 2 Automix			PANAVIA V5		
Immediate	One-day	TC 20k	Immediate	One-day	TC 20k	Immediate	One-day	TC 20k	Immediate	One-day	TC 20k
S	S	S	S	S	S	S	S	S	NS	S	S
PANAVIA SA Cement Universal			G-Cem ONE			ESTECEM II			SpeedCEM Plus		
Immediate	One-day	TC 20k	Immediate	One-day	TC 20k	Immediate	One-day	TC 20k	Immediate	One-day	TC 20k
S	S	S	S	S	S	S	S	S	NS	S	S
Variolink Esthetic DC			ResiCem EX			Nexus Universal Chroma					
Immediate	One-day	TC 20k	Immediate	One-day	TC 20k	Immediate	One-day	TC 20k			
NS	S	S	S	S	S	NS	S	S			

*n* = 10, Adh: number of adhesive failure modes after failure 5, TC 20k: after 20,000 thermocycles.

**Table 11:** Flexural modulus of resin cements [GPa, mean (S.D.)].

Resin Cement	Cure Mode	Time		
		Immediate	After One-Day Storage	TC 20k
RelyX Universal Resin Cement	Dual	1.31 (0.30)	4.27 (0.41)	4.24 (0.28)
	Self	0.33 (0.09)	2.84 (0.31)	3.81 (0.60)
RelyX Ultimate	Dual	4.74 (0.45)	9.22 (0.73)	9.40 (1.10)
	Self	0.44 (0.08)	4.52 (0.63)	5.39 (0.71)
RelyX Unicem 2 Automix	Dual	4.16 (0.51)	8.22 (0.58)	8.18 (0.69)
	Self	0.41 (0.07)	4.62 (0.80)	5.42 (0.52)
PANAVIA V5	Dual	0.65 (0.10)	6.76 (0.49)	5.89 (0.62)
	Self	0.52 (0.11)	2.92 (0.29)	3.44 (0.28)
PANAVIA SA Cement Universal (Automix)	Dual	1.04 (0.18)	5.18 (0.35)	5.73 (0.44)
	Self	0.40 (0.07)	3.26 (0.45)	3.62 (0.59)
G-Cem ONE	Dual	1.19 (0.20)	8.04 (0.60)	8.39 (1.02)
	Self	0.73 (0.16)	4.60 (0.69)	4.46 (0.58)
ESTECEM II	Dual	6.84 (0.91)	12.42 (1.79)	10.72 (0.67)
	Self	1.35 (0.35)	4.64 (0.80)	5.17 (0.55)
SpeedCEM Plus	Dual	2.31 (0.45)	5.82 (0.46)	5.36 (0.52)
	Self	0.62 (0.13)	3.66 (0.49)	3.69 (0.61)
Variolink Esthet DC	Dual	0.82 80.14)	6.36 (0.58)	5.48 (0.32)
	Self	0.81 (0.22)	4.53 (0.68)	4.50 (0.87)
ResiCem EX	Dual	3.28 (0.68)	7.82 (0.73)	9.27 (1.06)
	Self	0.96 (0.18)	4.78 (0.93)	4.81 (0.64)
Nexus Universal Chroma	Dual	1.37 (0.36)	8.17 (0.93)	7.69 (0.70)
	Self	0.75 (0.12)	3.53 (0.44)	3.84 (0.40)
Super-Bond Universal	Dual	NA	NA	NA
	Self	0.40 (0.06)	2.63 (0.43)	2.80 (0.21)

*n* = 10, TC 20k: after 20,000 thermocycles.

**Table 12:** Comparison of the means (Tukey HSD procedure) for flexural modulus of each resin cement material with regard to time [superscript letters (a–g) letters represent groups with no significant difference, *p* > 0.05].

RelyX Universal Resin Cement		RelyX Ultimate		RelyX Unicem 2 Automix		PANAVIA V5	
Dual-cure mode	Self-cure mode	Dual-cure mode	Self-cure mode	Dual-cure mode	Self-cure mode	Dual-cure mode	Self-cure mode
TC 20k ^a^	TC 20k ^b^	TC 20k	Immediate	Immediate	Immediate	Immediate ^f^	Immediate
Immediate ^a^	Immediate ^b^	Immediate ^c^	TC 20k	TC 20k ^d^	One-day ^e^	TC 20k ^f^	TC 20k ^g^
One-day	One-day	One-day ^c^	One-day	One-day ^d^	TC 20k ^e^	One-day ^f^	One-day ^g^
PANAVIA SA Cement Universal		G-Cem ONE		ESTECEM II		SpeedCEM Plus	
Dual-cure mode	Self-cure mode	Dual-cure mode	Self-cure mode	Dual-cure mode	Self-cure mode	Dual-cure mode	Self-cure mode
TC 20k ^a^	TC 20k ^b^	TC 20k	Immediate	Immediate	Immediate	Immediate ^f^	Immediate
Immediate ^a^	Immediate ^b^	Immediate ^c^	TC 20k	TC 20k ^d^	One-day ^e^	TC 20k ^f^	TC 20k ^g^
One-day	One-day	One-day ^c^	One-day	One-day ^d^	TC 20k ^e^	One-day ^f^	One-day ^g^
Variolink Esthetic DC		ResiCem EX		Nexus Universal Chroma		Super-Bond Universal	
Dual-cure mode	Self-cure mode	Dual-cure mode	Self-cure mode	Dual-cure mode	Self-cure mode	Dual-cure mode	Self-cure mode
TC 20k ^a^	TC 20k ^b^	TC 20k	Immediate	Immediate	Immediate	-	Immediate
Immediate ^a^	Immediate ^b^	Immediate ^c^	TC 20k	TC 20k ^d^	One-day ^e^	-	TC 20k ^g^
One-day	One-day	One-day ^c^	One-day	One-day ^d^	TC 20k ^e^	-	One-day ^g^

TC 20k: after 20,000 thermocycles.

**Table 13:** Comparison of means (Tukey HSD procedure) for flexural modulus of resin cement material at each time [superscript letters (a–f) represent groups with no significant difference, *p* > 0.05].

Immediate		After One-Day Storage		After TC 20k	
Dual-Cure Mode	Self-Cure Mode	Dual-Cure Mode	Self-Cure Mode	Dual-Cure Mode	Self-Cure Mode
PANAVIA V5 ^a^	RelyX Ultimate ^a^	RelyX Unicem 2 Automix ^a^	Nexus Universal Chroma ^a^	RelyX Unicem 2 Automix ^a^	Variolink Esthetic DC ^a^
Variolink Esthetic DC ^a b^	RelyX Unicem 2 Automix ^a b^	PANAVIA SA Cement Universal ^a b^	RelyX Universal Resin Cement ^a^	SpeedCEM Plus ^a b^	Nexus Universal Chroma ^a b^
PANAVIA SA Cement Universal ^a b^	PANAVIA SA Cement Universal ^a b^	SpeedCEM Plus ^a b c^	PANAVIA SA Cement Universal ^a^	RelyX Ultimate ^b c^	SpeedCEM Plus ^a b c^
Nexus Universal Chroma ^a b^	RelyX Universal Resin Cement ^a b^	RelyX Universal Resin Cement ^a b c^	RelyX Unicem 2 Automix ^a^	Variolink Esthetic DC^b c d^	ResiCem EX^a b c^
G-Cem ONE ^b^	SpeedCEM Plus ^a b c^		SpeedCEM Plus ^a^	G-Cem ONE ^b c d^	RelyX Ultimate ^b c d^
SpeedCEM Plus ^c^	Super-Bond Universal ^b c^	Variolink Esthetic DC ^c d^	RelyX Ultimate ^a b^	RelyX Universal Resin Cement ^c d^	RelyX Universal Resin Cement ^c d^
RelyX Universal Resin Cement ^d^	G-Cem ONE ^c^	G-Cem ONE ^d^	ResiCem EX ^b c^	PANAVIA SA Cement Universal ^c d e^	RelyX Unicem 2 Automix ^d e^
RelyX Ultimate ^d e^	Nexus Universal Chroma ^d^	Nexus Universal Chroma ^d^	G-Cem ONE ^b c^	Nexus Universal Chroma^d e^	Super-Bond Universal ^e^
RelyX Unicem 2 Automix ^d e^	Variolink Esthetic DC ^d^	ResiCem EX ^d^	ESTECEM II ^c^	PANAVIA V5 ^e^	G-Cem ONE ^e^
ResiCem EX ^e^	PANAVIA V5 ^d^	PANAVIA V5 ^e^	Variolink Esthetic DC ^c^	ResiCem EX ^e^	PANAVIA SA Cement Universal ^e f^
ESTECEM II ^f^	ResiCem EX ^e^	ESTECEM II ^f^	PANAVIA V5 ^c^	ESTECEM II ^f^	PANAVIA V5 ^f^
	ESTECEM II ^e^		Super-Bond Universal^c^		ESTECEM II ^f^

TC 20k: after 20,000 thermocycles.

**Table 14:** Comparison of the results (by *t*-test) for flexural modulus of each adhesive resin cement material with regard to two curing modes.

RelyX Universal Resin Cement			RelyX Ultimate			RelyX Unicem 2 Automix			PANAVIA V5		
Immediate	One-day	TC 20k	Immediate	One-day	TC 20k	Immediate	One-day	TC 20k	Immediate	One-day	TC 20k
S	S	NS	S	S	S	S	S	S	NS	S	S
PANAVIA SA Cement Universal			G-Cem ONE			ESTECEM II			SpeedCEM Plus		
Immediate	One-day	TC 20k	Immediate	One-day	TC 20k	Immediate	One-day	TC 20k	Immediate	One-day	TC 20k
S	S	S	S	S	S	S	S	S	S	S	S
Variolink Esthetic DC			ResiCem EX			Nexus Universal Chroma					
Immediate	One-day	TC 20k	Immediate	One-day	TC 20k	Immediate	One-day	TC 20k			
NS	S	S	S	S	S	S	S	S			

*n* = 10, Adh: number of adhesive failure modes after failure 5, TC 20k: after 20,000 thermocycles.

## Data Availability

The data presented in this study are available from the corresponding author, M.I., upon reasonable request.
